# The Antiarrhythmic Mechanisms of Flecainide in Catecholaminergic Polymorphic Ventricular Tachycardia

**DOI:** 10.3389/fphys.2022.850117

**Published:** 2022-03-09

**Authors:** Yukun Li, Xiaodong Peng, Rong Lin, Xuesi Wang, Xinmeng Liu, Rong Bai, Changsheng Ma, Ribo Tang, Yanfei Ruan, Nian Liu

**Affiliations:** ^1^Department of Cardiology, Beijing Anzhen Hospital, Capital Medical University, Beijing, China; ^2^National Clinical Research Center for Cardiovascular Diseases, Beijing, China; ^3^North China Medical and Health Group XingTai Hospital, Xingtai, China; ^4^Banner – University Medical Center Phoenix, University of Arizona College of Medicine, Phoenix, AZ, United States

**Keywords:** flecainide, antiarrhythmic mechanism, sodium channel, ryanodine receptor, catecholaminergic polymorphic ventricular tachycardia

## Abstract

Catecholaminergic polymorphic ventricular tachycardia (CPVT) is a severe yet rare inherited arrhythmia disorder. The cornerstone of CPVT medical therapy is the use of β-blockers; 30% of patients with CPVT do not respond well to optimal β-blocker treatment. Studies have shown that flecainide effectively prevents life-threatening arrhythmias in CPVT. Flecainide is a class IC antiarrhythmic drug blocking cardiac sodium channels. RyR2 inhibition is proposed as the principal mechanism of antiarrhythmic action of flecainide in CPVT, while it is highly debated. In this article, we review the current progress of this issue.

## Introduction

Catecholaminergic polymorphic ventricular tachycardia (CPVT) is a rare inherited arrhythmia syndrome characterized by bidirectional or polymorphic ventricular tachycardia (VT) provoked by emotional stress and/or physical activity. Clinical phenotypes include catecholamine-associated syncope and a characteristic pattern of bidirectional VT in the absence of structural heart disease (Leenhardt et al., 1995; Liu et al., 2008; Priori et al., 2013). The primary treatment strategy for CPVT is the use of β-blockers due to the catecholamine-dependent onset of VT, while insufficient protection from cardiac events has been reported despite optimal β-blockers therapy (Padfield et al., 2016; Yang et al., 2016). Flecainide, a classic antiarrhythmic agent, has been gaining the interest of clinicians in the treatment of CPVT. Accumulating clinical evidence shows that flecainide, alone and combined with β-blocker therapy, effectively prevents VT in patients with CPVT and has been recommended in the international guidelines (Liu et al., 2012; Steinfurt et al., 2015; Baltogiannis et al., 2019). In the initial study, the antiarrhythmic mechanism of flecainide in CPVT was the suppression of abnormal calcium release from the sarcoplasmic reticulum (SR) by targeting the cardiac ryanodine receptor (RyR2; Watanabe et al., 2009; Hilliard et al., 2010; Hwang et al., 2011). However, not all studies support this hypothesis (Liu et al., 2011; Sikkel et al., 2013; Bannister et al., 2015, 2016). In the last decade, the therapeutic mechanisms of flecainide in CPVT have become a major topic of debate in this field. In this review, we summarize and discuss the current progress in this field.

## Arrhythmogenic Mechanisms of CPVT

CPVT has been mainly related to mutations in genes encoding the cardiac ryanodine receptor (RyR2) and cardiac calsequestrin (CASQ2), which can be identified in 60–70% of CPVT patients (Wleklinski et al., 2020). RyR2 and CASQ2 are responsible for calcium homeostasis in cardiomyocytes.

The delicate balance of Ca^2+^ fluxes between the intracellular compartment and the extracellular space in cardiac myocytes is crucial for normal excitation-contraction (EC) coupling (Wier and Balke, 1999; Bers, 2002). During the plateau phase of the action potential, a small amount of Ca^2+^ enters the cytosol of cardiac myocytes *via* voltage-dependent L-type Ca^2+^ channels, resulting in a large amount of Ca^2+^ release into the cytosol *via* the RyR2 channel, which is called Ca^2+^-induced Ca^2+^ release (CICR). The cytosolic Ca^2+^ sharply from 150 nM to 1 μM activates the contractile apparatus. Then, the elevated cytosolic Ca^2+^ promptly resumed to 150 nM during the diastolic phase to ensure regular relaxation properties of the myocytes. The majority of Ca^2+^ is reuptake into the SR by Ca^2+^ ATPase isoform 2a (SERCA2a). The remaining Ca^2+^ is extruded into the extracellular fluid *via* a forward-mode Na^+^/Ca^2+^ exchanger (NCX).

The mutations of RyR2 and CASQ2 disrupt normal Ca^2+^ handling in the SR, enhancing the open probability of RyR2 and leading to spontaneous Ca^2+^ release events during the diastolic period. Under adrenergic stress, Ca^2+^ overload in the SR can facilitate abnormal Ca^2+^ leakage during relaxation. Elevated intracellular Ca^2+^ levels during the diastolic period would activate the forward mode of NCX, which extrudes Ca^2+^ in exchange for Na^+^ with a stoichiometry of 1:3, generating a net inward current. The transient inward current (Iti) produces delayed afterdepolarizations (DADs) and causes triggered activity once it reaches the threshold of the Na^+^ channel. Taken together, the molecular pathophysiology of arrhythmia occurrence in CPVT involves two critical steps: (1) spontaneous Ca^2+^ release from SR during the diastolic period, which could be exaggerated by adrenergic stimulation and (2) triggered activity activated by Iti, which is induced by spontaneous Ca^2+^ release.

## Clinical Efficacy and Safety of Flecainide in CPVT

The insufficient protection of β-blockers in CPVT has been reported (Padfield et al., 2016; Yang et al., 2016). Almost 30% of patients with CPVT still experience cardiac arrhythmias despite optimal β-blocker therapy. Therefore, it is important to explore alternative treatment options for CPVT. Knollman and his collaborators first reported that flecainide monotherapy or flecainide combined with β-blockers exhibited striking efficacy in preventing ventricular arrhythmias in two CPVT patients who did not respond to the combination therapy with β-blockers and verapamil (Watanabe et al., 2009). Subsequently, in a retrospective cohort study, the efficacy of flecainide was assessed in 33 patients with CPVT who were unprotected by conventional therapy (van der Werf et al., 2011). Ventricular arrhythmias were effectively controlled by flecainide in 22 patients (76%) and were completely suppressed in 14 patients (63%). In a randomized clinical trial, 14 patients with CPVT using maximally tolerated β-blockers demonstrated that ventricular arrhythmias during exercise were significantly reduced by flecainide, with complete suppression observed in 11 of 13 patients, and serious adverse events did not differ between the flecainide and placebo arms (Kannankeril et al., 2017). Table 1 lists the clinical efficacy and safety of flecainide treatment for CPVT in the literature (van der Werf et al., 2011; Khoury et al., 2013; Miyake et al., 2013; Watanabe et al., 2013; Roses-Noguer et al., 2014; Roston et al., 2015; Padfield et al., 2016; Kannankeril et al., 2017; Wangüemert Pérez et al., 2018). Overall, flecainide effectively prevented ventricular arrhythmias in patients with CPVT without apparent adverse events. Consequently, flecainide has been recommended for CPVT patients with ventricular arrhythmias who already have optimized β-blocker treatment.

**Table 1:** Clinical efficacy and safety of flecainide treatment in CPVT.

Study	Number of patients	Variants of CPVT	Combined β-blocker treatment	Daily dosage of flecainide	Follow-up	Cardiac events	Compliant	Side-effects
van der Werf et al., 2011	33	A4091T R2401H E4076K S4124G E4187Q E1724K R420W Y4962C G3946S R420Q R2474G F2215L R4157H M3978I V4771I	31 (94%)	150 mg (100–300; 1.5–4.5 mg/Kg)	20 months (12–40)	1/33 (39%) Appropriate ICD shock	No	6 (18%)
Watanabe et al., 2013	12	NR	12 (100%)	165 ± 46 mg (2.9 ± 1.3 mg/kg)	48 ± 94 months	2/11 (18%):1 SCD, 1 ACA	No	0 (0%)
Khoury et al., 2013	10	D307H	10 (100%)	200 mg (150–300 mg)	15.5 ± 10.4 months	2/10 (20%) appropriate ICD shocks(both preceded by supraventricular tachycardia)	Yes	0 (0%)
Miyake et al., 2013	3/24 (13%)	NR	3 (100%)	NR	29 (7–82) months	0	NR	No
Roses-Noguer et al., 2014	7/13 (53%)	NR	7 (100%)	2.3 ± 1 mg/kg	4.0 years (1.7–19.9)	2/7 (29%)appropriate ICD shock	NR	No
Roston et al., 2015	51/211 (24%)	E243K R4959Q V4471I	43 (96%)	NR	1.3 years (0.9–2.7)	8 (16%; suboptimal dose)	No (6/8)	5 (10%)
Padfield et al., 2016	8	T259_A283 S4124G R420W	0 (0%)	150 mg (100–200 mg)	37.1 months (1.4–75.5)	0	NR	1 (12.5%)
Kannankeril et al., 2017	13	NR	13 (100%)	0.50–0.80 μg/ml (target serum level)	3 months	2/13 (15%)Persistent bigeminy	Yes	0 (0%)
Wangüemert Pérez et al., 2018	18/174 (10%)	G357S Thr415lle Gly4140Glu lle4857Asn Ala157Ser	17 (94%)	159.38 mg (2.3 mg/kg/d).	2.63 ± 1.28 years	2/18 (11%):1 appropriate ICD shock 1 syncopal episode	NR	0 (0%)

ICD = implantable cardioverter defibrillator; NR = not reported.

## Flecainide Prevents Arrhythmias by Targeting RyR2

The abnormal Ca^2+^ leak events from RyR2 are the essence of molecular arrhythmogenic mechanism in CPVT. Theoretically, direct RyR2 blockers are promising mechanism-based therapies for CPVT. Tetracaine, a sodium channel blocker, is a RyR2 blocker that effectively inhibits Ca^2+^ leak from SR. Thus, Knollmann and his collaborators screened the RyR2 inhibiting effects of clinically available sodium channel blockers in a lipid bilayer study and found that flecainide reduced the duration of RyR2 channel openings, but not its closed channel duration. They then tested the effects of flecainide on CASQ2 knockout mice. Intraperitoneal administration of flecainide completely suppressed exercise-induced VT *in vivo*, and incubation with flecainide significantly ameliorated the spontaneous Ca^2+^ release from SR induced by isoproterenol in isolated myocytes. In contrast, without RyR2 blocking action in the lipid bilayer, lidocaine did not show therapeutic effects *in vivo* and *in vitro*. Therefore, they propose that the underlying antiarrhythmic mechanism of flecainide in CPVT attributes to its RyR2 blockade but not its intrinsic sodium channel inhibiting action.

Subsequently, the Knollman group performed a series of experiments to reinforce the concept. Galimberti and Knollmann (2011) reported that flecainide suppressed the spontaneous Ca^2+^ wave with IC_50_ 12.8 μM in permeabilized ventricular myocytes. The blocking action of flecainide is use-dependent, suggesting that RyR2 activity determines the potency and efficacy of flecainide. Given flecainide’s blocking features of the sodium channel and RyR2 channel, it is challenging to dissect the antiarrhythmic mechanisms of flecainide in CPVT. Kryshtal et al. (2021) synthesized a flecainide analogues, named N-methyl flecainide, which has the sodium channel blocking action but without the RyR2 inhibiting effect. They reported that flecainide, but not N-methyl flecainide, significantly reduced arrhythmias in CPVT transgenic mice and decreased spontaneous calcium-release events in intact and membrane-permeabilized myocytes. Therefore, they concluded that RyR2 channel inhibition, but not sodium channel blockade, is likely the principal mechanism of the antiarrhythmic action of flecainide in CPVT.

## Flecainide Prevents Arrhythmias by Targeting the Sodium Channel

Flecainide is a hydrophilic sodium channel blocker with a pKa of 9.3. At pH 7.4, only 1% of flecainide is neutral and is available for diffusion across the membrane of myocytes (Liu et al., 2012). The intrinsic feature of flecainide makes it difficult to quickly achieve sufficient concentration to block RyR2, which is located in the intracellular space. Thus, the RyR2 blocking action of flecainide cannot fully explain the rapid amelioration of spontaneous Ca^2+^ release after the acute administration of flecainide in isolated cardiac myocytes.

**Figure 1:**
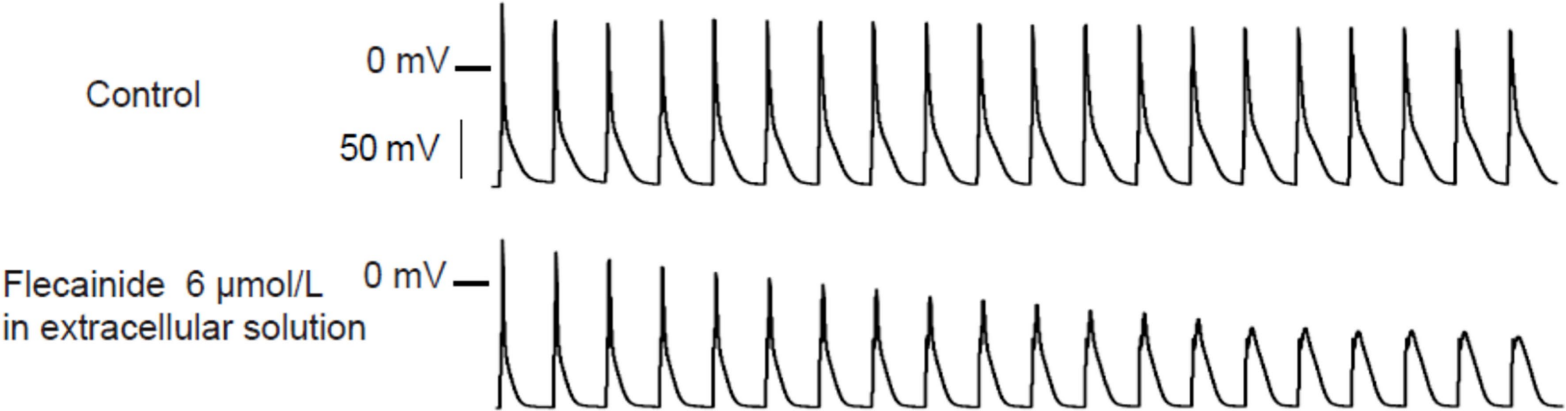
Representative action potential recordings of RyR2^R4496C+/^ myocytes at 5 Hz pacing. Flecainide (6 μM) almost abolished the upstroke of the action potential. Reproduced with permission by Lippincott Williams and Wilkins from Liu et al. (2011).

**Figure 2:**
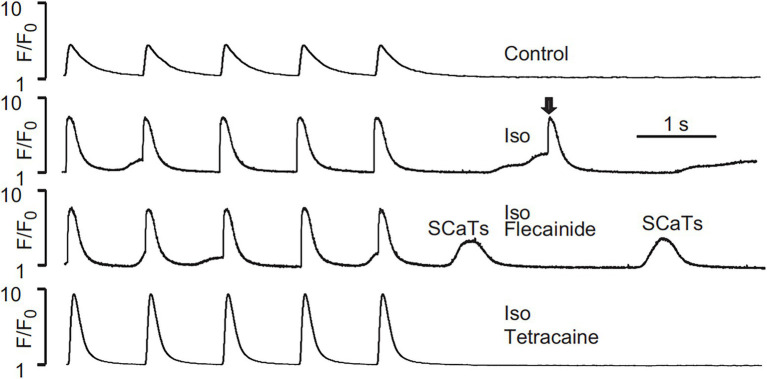
SCaTs elicited in an RyR2^R4496C+/−^ myocyte in the presence of isoproterenol (Iso) at 1 Hz pacing. The arrow shows a triggered SCaT, presumably from a triggered electric beat induced by SCaTs. Flecainide (incubation for 30 min) did not prevent isoproterenol-induced SCaTs in an RyR2^R4496C+/−^ myocyte. Reproduced with permission by Lippincott Williams and Wilkins from Liu et al. (2011).

Liu et al. (2011) tested the effects of flecainide in a CPVT RyR2-R4496C^+/−^ mouse model. Flecainide significantly reduced ventricular arrhythmias induced by adrenaline and caffeine *in vivo*. In isolated intact RyR2^R4496C+/−^ myocytes, flecainide did not affect Ca^2+^ transient amplitude, decay, or SR Ca^2+^ content. In permeabilized RyR2^R4496C+/−^ myocytes, flecainide did not alter the frequency of spontaneous Ca^2+^ sparks. In contrast, when the dosage of flecainide reached 6 μM, the upstroke of action potential was blunted significantly at the pace of 5 Hz (Figure 1). Flecainide effectively prevented isoproterenol-induced triggered activity but had little effect on spontaneous Ca^2+^ transients (SCaTs) elicited by isoproterenol (Figure 2). The threshold for action potential induction increased significantly after acute administration of flecainide. Based on the above data, Liu et al. suggested that the antiarrhythmic mechanism of flecainide was mediated by its Na^+^ channel blockade.

Sikkel et al. (2013) performed a study to explore the effects of flecainide on Ca^2+^ handling in isolated rat ventricular myocytes. They found that sodium channel blockers (flecainide, tetrodotoxin, propafenone, and lidocaine) could reduce spontaneous Ca^2+^ release events under their experimental conditions. After inactivation of the sodium channel using the voltage-clamp approach, flecainide could not reduce Ca^2+^ waves. Therefore, they proposed that Na^+^ channel blockade by flecainide could reduce Na^+^ influx into cardiac myocytes, resulting in the enhancement of Ca^2+^ efflux through NCX and decrease of Ca^2+^ in the vicinity of the RyR2 channels, ultimately reducing the frequency of spontaneous Ca^2+^ release events. In the HEK293 cell line expressing hRyR2, which is devoided of Na^+^ channels in the cellular membrane, Bannister et al. reported that flecainide did not affect spontaneous Ca^2+^ release events (Bannister et al., 2015, 2016). Thus, these studies suggest that flecainide’s antiarrhthymic action in CPVT relies on its Na^+^ channel blockade but not RyR2 inhibition.

Neuronal sodium channels are expressed in the cellular membranes of cardiac myocytes. Radwański et al. (2015a) demonstrated that 100 nM TTX, which blocks neuronal sodium channels but not NaV1.5, significantly reduced and desynchronized spontaneous Ca^2+^ release events in isolated myocytes. Next, they demonstrated that the NaV1.6 blocker riluzole ameliorated spontaneous Ca^2+^ release events *in vitro* and reduced arrhythmias *in vivo* in a CPVT mouse model. Cardiac Na^+^ and Ca^2+^ cycling interplay in the nanodomains beneath the membrane. They speculated that targeting neuronal sodium channels may be a promising therapeutic strategy for Ca^2+^ dysregulation-associated heart diseases such as CPVT and heart failure, which would not compromise electrical excitability, which is proarrhythmic (Radwański et al., 2015b).

## Dissecting the Antiarrhythmic Mechanisms of Flecainide in CPVT

There is a hot debate on the antiarrhythmic mechanisms of flecainide in CPVT. The critical issue is whether flecainide is a RyR2 blocker, as the major mechanism responsible for the efficacy of flecainide in CPVT has been observed in clinical practice. There are multiple targets of flecainide in cardiac myocytes, including sodium channel, potassium channel, and RyR2 channel et al. (Table 2). It is challenging to dissect the therapeutic mechanisms of flecainide in intact cardiac myocytes because of its influence on Na^+^, Ca^2+^, and K^+^ homeostasis at the cellular level simultaneously.

**Table 2:** Blocking effects of flecainide on ion channels.

Species	Channel	Current	Reference
Human	Nav1.5	INa	Ramos E, J Physiol. 560(Pt 1):37–49
	RyR2	Cytoplasm – SR	Bannister ML, Circ Res. 116(8):1324–35
	Kv11.1	IKr	Melgari D, J Mol Cell Cardiol. 86:42–53
	Kv4.3	Ito1	Paul AA, Br J Pharmacol. 136(5):717–29
	Kv1.5	IKur	Herrera D, Mol Pharmacol. 68(2):305–16
Sheep	RyR2	Cytoplasm – SR	Hilliard FA, J Mol Cell Cardiol. 48(2):293–301
	RyR2	SR – Cytoplasm	Mehra D, Mol Pharmacol. 86(6):696–706
Dog	Kv1.5	IKur	Yue L, Cardiovasc Res. 46(1):151–61
	Kv3.1	IKur	Herrera D, Mol Pharmacol. 68(2):305–16
Rabbit	HCN4	If	Tamura A, J Pharmacol Sci. 110(2):150–59

Single RyR2 channel experiments in artificial lipid bilayers appear to resolve this issue directly. The initial study by Knollmann et al. tested the inhibitory potency of flecainide on the current flow in the cytoplasm-to-lumen direction in sheep RyR2 channels and found that it inhibited the duration of channel openings and did not affect closed channel duration (Watanabe et al., 2009). The reduction in open-channel probability was concentration-dependent, with an IC_50_ of 55 ± 8.1 μM. Later, this group presented a detailed analysis of the kinetics of RyR2 inhibition by flecainide. Flecainide inhibited RyR2 by two distinct modes: a fast block consisting of brief substrate and closed events with a mean duration of ∼1 ms, and a slow block consisting of closed events with a mean duration of ∼1 s (Mehra et al., 2014). These two modes are independent mechanisms for RyR2 inhibition.

Under physiological conditions, the current flow of cardiac RyR2 is directed from the lumen to the cytoplasm. Bannister et al. (2015) tested the effects of flecainide on the luminal-to-cytosolic flux of cations through human RyR2 in a lipid bilayer study and reported that flecainide, even at supraphysiological concentrations, did not inhibit the open probability of RyR2. Since the ion fluxes across the SR membrane are bidirectional, the authors also explored whether flecainide modulates cytoplasm-to-SR luminal “countercurrent.” They found that 50 μM flecainide had a negligible effect on the mechanisms responsible for the SR charge-compensating counter current (Bannister et al., 2015, 2016). More recently, the study by Salvage et al. showed that low cytoplasmic concentrations (0.5–10 μM) of flecainide activated isolated mouse RyR2 channels. In contrast, high cytoplasmic concentrations (50–100 μM) of flecainide showed an inhibitory action (Salvage et al., 2021).

RyR2 is a macro-molecular complex, and numerous accessory proteins modulate RyR2’s function, such as FKBP12, FKBP12.6, calmodulin, and S100A1 (Ritterhoff et al., 2014, 2015). Flecainide may affect RyR2’s function directly or indirectly by binding to accessory proteins. The procedure for purifying RyR2 for single-channel recording may disrupt the interaction between accessory proteins and RyR2. This might be an interpretation of the controversial results from different laboratories.

Permeabilized myocytes, devoid of the influence of the cellular membrane, allow direct exploration of the effects of flecainide on Ca^2+^ handling in SR. Hilliard et al. (2010) demonstrated that 25 μM flecainide significantly reduced the spontaneous Ca^2+^ wave frequency in permeabilized ventricular myocytes. Savio-Galimberti and Knollmann (2015) showed that flecainide suppressed spontaneous Ca^2+^ wave with IC_50_ 15.6 ± 3.4 μM in permeabilized CASQ2^−/−^ myocytes. On the contrary, in permeabilized RyR2^R4496C^ myocytes, Liu et al. (2011) reported that 6 μM flecainide did not affect the frequency of spontaneous Ca^2+^ sparks; Bannister et al. (2015) failed to confirm the reduction of spontaneous Ca^2+^ wave frequency after administration of 25 μM flecainide in permeabilized rat cardiac myocytes. However, it is difficult to reconcile these conflicting results. Permeabilized myocytes are indispensable for disrupting the intracellular structure and loss of cytosolic proteins, which depend on the degree of membrane permeabilization induced by the concentration of saponin or β-escin and the duration of exposure to the agent. This may differ among laboratories, leading to controversial results (Smith and MacQuaide, 2015).

Flecainide has a narrow therapeutic window between the effective dose and the dose that can produce adverse toxic effects. The target range for flecainide concentration is 0.5–2.4 μM in the clinical practice (Melgari et al., 2015; Rabêlo Evangelista et al., 2021; Yang et al., 2021). Despite conflicting results in single-channel recordings, the therapeutic concentration of flecainide cannot block RyR2 effectively. Based on the IC_50_ of flecainide for inhibiting RyR2, a high flecainide concentration (25 μM) was used in intact or permeabilized myocytes to elucidate antiarrhythmic mechanisms. Watanabe et al. (2009) reported that flecainide concentration in cardiac tissue is 33 ± 0.8 μM 1 h after injection in mice, suggesting that flecainide can accumulate in cardiac tissue. Thus, it seems reasonable to use very high concentrations of flecainide in the experiments. In a study by Liu et al. (2011), 6 μM flecainide completely abolished the upstroke of action potentials in mouse ventricular myocytes, consistent with the adverse toxic effects of high-dose flecainide administration. In this scenario, it is unlikely that the blockade of RyR2 by high concentrations of flecainide (25 μM) is the primary mechanism underlying its dramatic efficacy in CPVT (Liu et al., 2011). Moreover, despite the high concentration of flecainide in cardiac tissue, it cannot be arbitrarily inferred that flecainide in the cytoplasm of cardiac myocytes reaches a sufficient concentration to block RyR2. It is almost impossible for high concentrations of flecainide in cardiac tissue to exclusively affect RyR2, but not cardiac sodium channels.

Flecainide is a multiple potassium channel blocker. Class III antiarrhythmic drugs are effective in the reentrant arrhythmias but not trigger arrhythmias. They are not effective in CPVT in the clinical setting. Potassium channel blockers prolong the action potential duration, leading to Ca^2+^ overload, and are detrimental to CPVT (Němec et al., 2010). In this scenario, potassium channel block is unlikely responsible for the flecainide efficacy in CPVT.

Most of the studies in CPVT are from small animal models, and mouse has a small heart size and fast heart rate. We need to be aware of the limitations to generalize the results of the small animal research to humans (Joukar, 2021).

## Sodium Channel Blockers Treat CPVT Phenocopy

Bidirectional VT is a typical arrhythmic phenotype observed in patients with CPVT (van der Werf et al., 2012). It has been proposed that bidirectional VT in CPVT, digitalis toxicity, and Andersen-Tawil Syndrome (ATS) share a similar underlying electrophysiological mechanism, which is associated with alternating ectopic foci originating from the distal His-Purkinje system in the left and/or right ventricle, induced by Ca^2+^ overload in Purkinje cells (Smith et al., 2006; Tristani-Firouzi and Etheridge, 2010).

Digitalis intoxication is manifested by Na^+^-K^+^ pump inhibition, resulting in intracellular Ca^2+^ overload, which causes triggered arrhythmias, such as bidirectional VT. The sodium channel blockers lidocaine and phenytoin have been recommended for the effective treatment of dysrhythmias associated with digitalis intoxication (French et al., 1984; Antman and Smith, 1985). In isolated myocytes, sodium channel blockers can ameliorate intracellular Ca^2+^ overload induced by digitalis and reduce spontaneous Ca^2+^ release events. Since there is no RyR2 blocking action of lidocaine and phenytoin, the sole sodium channel blockade is responsible for the antiarrhythmic effects of lidocaine and phenytoin in digitalis intoxication.

ATS, which is mainly caused by KCNJ2 mutations, phenocopies CPVT and may manifest the typical adrenergically mediated bidirectional VT (Zhang et al., 2005). The underlying arrhythmogenic mechanism is triggered arrhythmias induced by Ca^2+^ dysregulation in cardiac myocytes. A series of cases have shown that flecainide effectively prevents arrhythmias in patients with ATS (Pellizzón et al., 2008; Kuroda et al., 2017). Given that ATS presents bidirectional VT, which is usually observed in digitalis toxicity, and phenytoin is used to treat arrhythmia in digitalis toxicity, Maneesh et al. tested phenytoin in three ATS patients who did not respond to conventional therapy (β-blockers, flecainide, and verapamil). They reported that phenytoin completely suppressed ventricular arrhythmias in two patients and significantly reduced ventricular arrhythmias burden in one patient (Rai et al., 2019). Thus, sodium channel inhibition is likely the principal mechanism of flecainide action in ATS.

Another interesting issue is the diverse responses to sodium channel blockers in CPVT. Use and frequency dependence are common properties of class I antiarrhythmic agents. Flecainide, but not lidocaine, preferentially blocks sodium channel in the open state (Kojima et al., 1989; Ramos and O’Leary, 2004). The electrophysiological mechanism of CPVT is that DAD reaches the threshold of Na^+^ channel and results in triggered activity, which is also frequency-dependent. Therefore, flecainide is more effective than other sodium channel blockers during fast heart rates, such as conditions in which patients with CPVT develop cardiac arrhythmias. In CPVT, the efficacy of other sodium channel blockers with the strong use dependence block, such as propafenone and pilsicainide, needs further investigation.

## Flecainide Treatment in Calcium-Release Deficiency Syndrome

Recently, loss of function (LOF) of RyR2 mutations has been identified as a new clinical entity, termed cardiac ryanodine receptor calcium-release syndrome (CRDS), which is characterized by ventricular fibrillation and sudden death. However, it does not manifest ventricular tachyarrhythmias during stress testing (Roston et al., 2021). CRDS is a mirror image of CPVT due to the opposite of the RyR2 function. It is logical to infer that flecainide might exacerbate the CRDS phenotype if it can inhibit RyR2. To date, flecainide has proven to be a promising therapeutic agent for CRDS (Tester et al., 2020; Ormerod et al., 2021). The programmed electrical stimulation protocol with a pattern of long-burst, long-pause, and short-coupled (LBLPS) can induce ventricular arrhythmias in transgenic mice with RyR2 LOF mutations. Sun et al. (2021) demonstrated that treatment with flecainide abolished LBLPS-induced ventricular arrhythmias in model mice. In the induced pluripotent stem cell cardiomyocytes carrying homozygous RYR2 duplication, which presented LOF, Tester et al. (2020) reported that flecainide significantly reduced arrhythmic activity caused by isopropanol. Ormerod et al. (2021) tested flecainide in nine CRDS patients and found that the administration of flecainide substantially reduced arrhythmia inducibility in one subject and abolished arrhythmia in all others. Sun et al. (2021) proposed that the therapeutic mechanisms of flecainide in CRDS are attributable to its multiple blocking of membrane channels.

## Summary

Flecainide has a significant impact on the clinical management of patients with CPVT. Efforts have been made to explore the underlying mechanisms of flecainide therapy for CPVT. There is a hot debate regarding the effects of flecainide on RyR2. Understanding the mechanisms of flecainide in CPVT will improve our knowledge of Ca^2+^ dysregulation in cardiac myocytes and help develop a more specific therapeutic strategy for CPVT.

## Author Contributions

YR and NL defined the theme of review. YL wrote the manuscript. XP, RL, XW, and XL took part in preparing the manuscript. YR, NL, RT, CM, and RB prepared and reviewed the manuscript before publication. All authors confirmed that they have read and approved the manuscript and they have met the criteria for authorship.

## Funding

This work was supported by the National Science Foundation of China (grant nos. 81770318, 82170318, and 81870244) and Beijing Municipal Natural Science Foundation (grant no. 7192051).

## Conflict of Interest

The authors declare that the research was conducted in the absence of any commercial or financial relationships that could be construed as a potential conflict of interest.

## Publisher’s Note

All claims expressed in this article are solely those of the authors and do not necessarily represent those of their affiliated organizations, or those of the publisher, the editors and the reviewers. Any product that may be evaluated in this article, or claim that may be made by its manufacturer, is not guaranteed or endorsed by the publisher.
